# Hyperbranched Polyglycerol as a Colloid in Cold Organ Preservation Solutions

**DOI:** 10.1371/journal.pone.0116595

**Published:** 2015-02-23

**Authors:** Sihai Gao, Qiunong Guan, Irina Chafeeva, Donald E. Brooks, Christopher Y. C. Nguan, Jayachandran N. Kizhakkedathu, Caigan Du

**Affiliations:** 1 Department of Urologic Sciences, the University of British Columbia, Vancouver, BC, Canada; 2 Department of Thoracic and Cardiovascular Surgery, Tongji Hospital, Huazhong University of Science and Technology, Wuhan, P.R. China; 3 Centre for Blood Research, and the Department of Pathology and Laboratory Medicine, University of British Columbia, Vancouver, BC, Canada; 4 Department of Chemistry, University of British Columbia, Vancouver, BC, Canada; Imperial College London, Chelsea & Westminster Hospital, UNITED KINGDOM

## Abstract

Hydroxyethyl starch (HES) is a common colloid in organ preservation solutions, such as in University of Wisconsin (UW) solution, for preventing graft interstitial edema and cell swelling during cold preservation of donor organs. However, HES has undesirable characteristics, such as high viscosity, causing kidney injury and aggregation of erythrocytes. Hyperbranched polyglycerol (HPG) is a branched compact polymer that has low intrinsic viscosity. This study investigated HPG (MW-0.5 to 119 kDa) as a potential alternative to HES for cold organ preservation. HPG was synthesized by ring-opening multibranching polymerization of glycidol. Both rat myocardiocytes and human endothelial cells were used as an *in vitro* model, and heart transplantation in mice as an *in vivo* model. Tissue damage or cell death was determined by both biochemical and histological analysis. HPG polymers were more compact with relatively low polydispersity index than HES in UW solution. Cold preservation of mouse hearts *ex vivo* in HPG solutions reduced organ damage in comparison to those in HES-based UW solution. Both size and concentration of HPGs contributed to the protection of the donor organs; 1 kDa HPG at 3 wt% solution was superior to HES-based UW solution and other HPGs. Heart transplants preserved with HPG solution (1 kDa, 3%) as compared with those with UW solution had a better functional recovery, less tissue injury and neutrophil infiltration in syngeneic recipients, and survived longer in allogeneic recipients. In cultured myocardiocytes or endothelial cells, significantly more cells survived after cold preservation with the HPG solution than those with the UW solution, which was positively correlated with the maintenance of intracellular adenosine triphosphate and cell membrane fluidity. In conclusion, HPG solution significantly enhanced the protection of hearts or cells during cold storage, suggesting that HPG is a promising colloid for the cold storage of donor organs and cells in transplantation.

## Introduction

Cold preservation of donor organs, tissues and cells with a specialized solution is a common strategy to minimize ischemic injury prior to transplantation, and so far many different preservation solutions have been developed for this purpose [1]. In heart transplantation, the University of Wisconsin (UW) solution has been demonstrated to be a safe and effective preservation solution for heart transplants [2–4], and is associated with improved short-term survival and less acute ischemic necrosis in the early post-transplant period [5–7]. One of the important components in UW solution, ET-Kyoto solution [8] and KPS-1 solution [1], is hydroxyethyl starch pentafraction (HES). Inclusion of HES is essential to counteract graft interstitial edema and endothelial cell swelling for improving viability and outcomes of donor organ preservation [9, 10], but it may have negative effects on its application as a cold organ preservation solution for transplantation. HES is commonly used for volume resuscitation of critically ill patients, but its safety has recently come into question. Fluid resuscitation with HES is associated with coagulopathy, pruritus and acute kidney injury [11–15], and HES impairs immediate donor kidney function after transplantation [16]. Two other important limitations associated with HES in UW solution have also been suggested: (i) it increases the viscosity of the solution, resulting in an increase in vascular resistance of donor organs to the initial flushing/perfusion that could shorten graft survival [17, 18]; and (ii) it causes the hyperaggregation of human red blood cells (RBCs) due to the adsorption of HES to RBCs [19–21], which may result in stasis of blood and incomplete washout from donor organs before transplantation. Moreover, HES is a highly heterogeneous polymer with high polydispersity index (PDI). Therefore, there is an unmet need for an alternative colloid that is superior to HES in terms its biocompatibility as well as for maximally limiting donor organ injury during organ procurement or preservation.

In addition to HES, both dextran and polyethylene glycol (PEG) are used as colloids in new organ preservation solution development [1]. Examples include dextran-40 in low-potassium dextran (LPD) solution [22], PEG-35 in institut georges lopez (IGL)-1 solution [23], and PEG-20 in SCOT-15 [24]. However, all these polymers including HES have high intrinsic viscosities owing to their large hydrodynamic radii and predominantly linear polymer structures. Just like HES [19–21], high molecular weights (MW) of both dextran and PEG have been reported to aggregate RBCs at the high concentrations [25–27], and the adsorption of these polymers on the cell surface is also higher [27]. In general, branched polymers are more compact than linear polymers, leading to smaller hydrodynamic sizes and low solution viscosities or less resistance to flow [28–32]. Therefore, a compact branched polymer may have advantages over linear polymers when used in an organ preservation setting, where high viscosity and adsorption to red blood cells and vascular endothelial lining are not desirable, during flushing and storage of donor organs at cold temperature.

**Fig 1:**
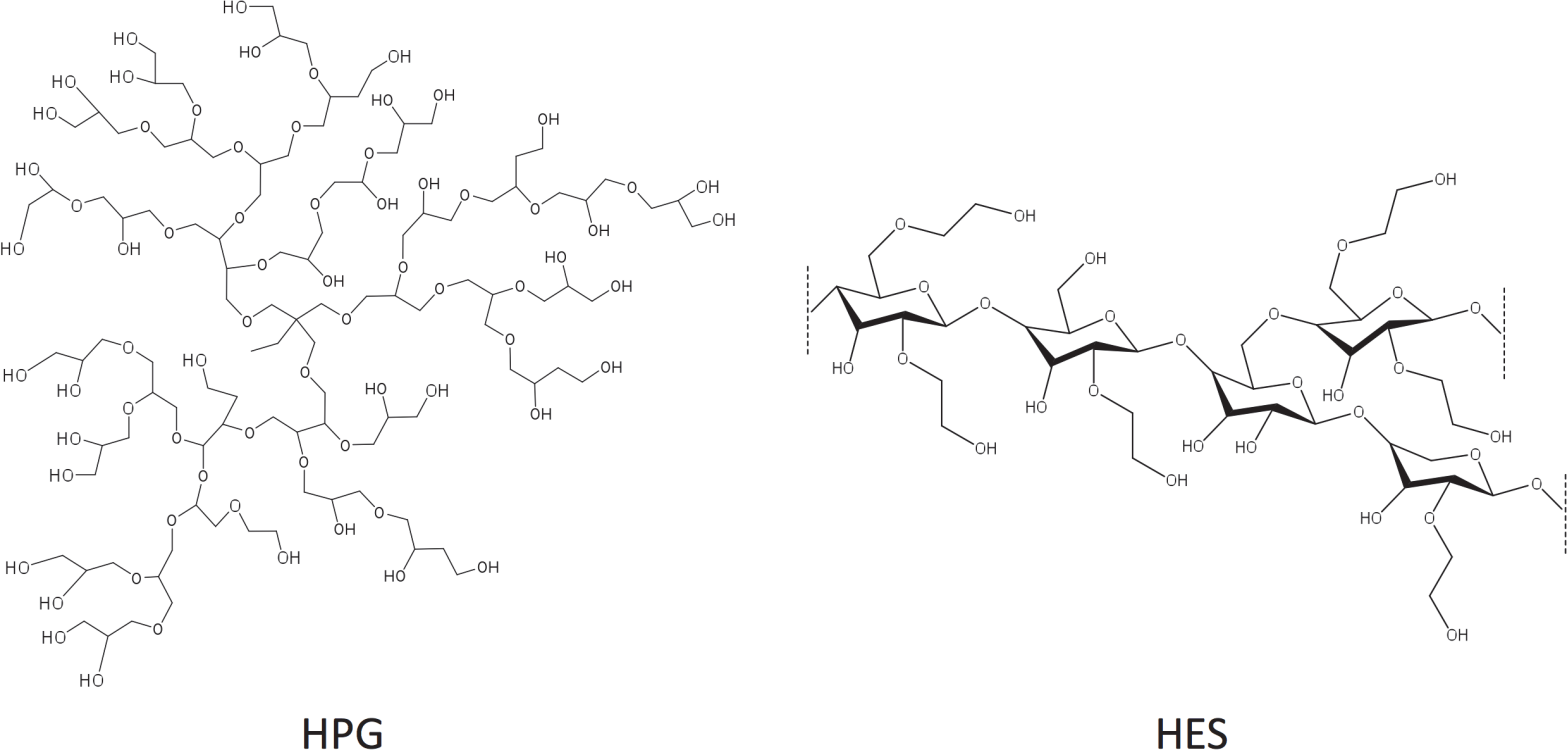
Chemical structure of HPG or HES.

Hyperbranched polyglycerol (HPG) is a branched polyether polymer with multiple hydroxyl functionalities (Fig. 1), and can be easily prepared by an overnight reaction of ring-opening multibranching polymerization (ROMP) of glycidol with good control over MW and low PDI [33–35]. It has been well documented that HPG is blood compatible, has low solution viscosity, and is non-immunogenic and non-toxic in mice [34, 36–41]. The potential of this polymer for medical application has been actively investigated in many fields, including: restoration of circulating volume [38], drug delivery [42, 43], antigen protection on red blood cells (RBCs) [44–46] and as an osmotic agent in peritoneal dialysis solution [47, 48].

The following facts suggest that HPG is a viable polymer colloid in the cold preservation of donor organs. HPG is a highly water soluble (> 400 mg/mL), compact polymer with equal or better biocompatibility profile than PEG (an FDA approved polymer). HPG has low intrinsic viscosity similar to that of proteins, approximately 10-times lower than those of linear polymers of equivalent MW (i.e. PEG, HES, dextran) [38, 41]. Furthermore, unlike linear polymers, HPG neither precipitates proteins nor aggregates cells (e.g. RBCs) even at very high concentrations [41, 44, 45, 49]. All of these properties suggest that HPG may be superior to HES, dextran or PEG for the development of a novel preservation solution, that can significantly reduce the resistance of the organ to cold flushing/perfusion and also provide better protection in storage. Thus in this study, the efficacy of the HPG-based solution in hypothermic preservation of mouse hearts, human endothelial cells, myocardiocytes was investigated as compared with HES-based UW solution.

## Materials and Methods

### Ethics Statement

This study was carried out in strict accordance with the recommendations in the Guide for the Care and Use of Laboratory Animals of the Canadian Council on Animal Care. The protocol was approved by the Committee on the Ethics of Animal Experiments of the University of British Columbia (Permit Number: A09-0273). All surgery was performed under ketamine/xylazine/isoflurane anesthesia, and all efforts were made to minimize suffering.

### Reagents

All the laboratory general reagents and chemicals were purchased from Sigma-Aldrich Canada (Oakville, ON, Canada) and used without further purification unless mentioned. Glycidol was purified by distillation under reduced pressure before use and stored over molecular sieves at 4°C.

### Polymer Synthesis and Characterization

Different MWs of HPG were synthesized by anionic ROMP of glycidol as described previously [33]. A typical protocol of HPG synthesis was described recently [47]. A high MW of HPG (119 kDa) was synthesized following a recently developed procedure from our laboratory [35]. The newly synthesized polymer was precipitated in acetone and dried, and then dissolved in water and dialyzed against deionized water. The final solution was lyophilized to recover the polymer. The MW of each polymer was determined by using Gel Permeation Chromatography (GPC) along with Waters ultrahydrogel columns and 0.1 N NaNO_3_ as an eluent. A dn/dc of 0.12 mL/g was used for the MW determination. The chemical structure and branching of different HPGs were characterized by proton NMR and ^13^C NMR analysis [35]. The average hydrodynamic size (Rh) of the HPG was measured using quasi elastic light scattering (QELS) detector coupled to the MALLS detector from Wyatt technology (Santa Barbara, CA, USA). The MW of HES was determined using GPC-MALLS. The UW solution was dialyzed against water (MWCO membrane-3.5 kDa), and the polymer was recovered by lyophilisation. The HES was dissolved in 0.1 N NaNO_3_ solution and injected. A dn/dc of 0.145 mL/g was used for the MW determination.

### Preparation of HPG Preservation Solutions

HPG-based preservation solutions were prepared by dissolving HPG (w/v) in a solution containing: 100 mM lactobionic acid, 100 mM potassium hydroxide (KOH), 25 mM potassium dihydrogen phosphate (KH_2_PO_4_), 5 mM magnesium sulfate (MgSO_4_), 5 mM adenosine, 3 mM glutathione, and 1 mM allopurinol, the same composition as in UW solution (DuPont Canada, Mississauga, ON, Canada) omitting 30 mM raffinose and 5% HES. The pH of HPG preservation solutions was adjusted to 7.4 using NaOH/HCl at 22°C. The osmolality of the solutions was determined using The Advanced Osmometer Model 3D3 (Advanced Instruments, Inc, MA, USA). The relative viscosity of the HPG and UW solutions at different temperatures was measured using an Ubbelohde viscometer (Cannon-Manning 50, A 412 Semi-Micro Viscometer, sample volume approximately 1.0 mL, CANNON Instrument Company, PA 16803, USA).

### Animals and Cell Culture

C57BL/6j (B6, H-2^b^) and BALB/c mice (H-2^d^) (males, 8–10 weeks old) were purchased from the Jackson Laboratory (Bar Harbor, ME, USA). All procedures related to animal use in this study were performed and monitored in accordance with the Canadian Council on Animal Care guideline under the protocols approved by the Animal Use Subcommittee at the University of British Columbia. Human umbilical vein endothelial cells (HUVECs) and cell culture conditions were described previously [50], and H9c2 cells (CRL-1446, ATCC, Manassas, VA, USA), a rat cardiomyocyte cell line, were grown in Dulbecco’s Modified Eagle’s Medium (DMEM) supplemented with 4 mM L-glutamine, 10% fetal bovine serum (FBS) and 100 U/mL penicillin-streptomycin at 37°C in a humidified atmosphere of 5% CO_2_.

### Donor Preservation and Heterotopic Cardiac Transplantation

Donor hearts were harvested from B6 donor mice after perfusion with 10 units/mL of heparin, and stored with ligated pulmonary veins in either the HPG solution or UW solution at 4°C. After 24 h of cold preservation, the donor hearts were heterotopically transplanted into either syngeneic B6 mice (B6 to B6 isotransplantation) or allogeneic BALB/c mice (B6 to BALB/c allotransplantation) as described previously [51]. The 24 h of prolonged cold preservation was designed in order to reveal the differences easily between HPG solutions and control—UW solution in organ protection. The graft function was scored by its contraction or beating at both 15 min and 24 h after graft transplantation according to a semi-quantitative method as described previously [51, 52]. In allotransplantation, the recipient mice received cyclosporine (CsA) therapy (15 mg/kg/day, Novartis, Basel, Switzerland) immediately after surgery to prevent graft rejection until the cessation of graft beat or for 20 days. Graft survival was assessed by daily transabdominal palpation in a blinded fashion. Cessation of graft beat indicated the failure of heart transplant, which was subsequently confirmed by histological examination.

### Measurements of Creatine Kinase (CK) and Lactate Dehydrogenase (LDH)

The severity of heart tissue damage or cell death with cell membrane disruption was determined by the release of intracellular LDH (a marker for cell death) and/or CK (a specific marker for myocardial tissue damage) to the preservation solution or serum. LDH release was quantitated by the LDH assay using a cytotoxicity detection (LDH) kit (Roche Applied Science, Laval, QC, Canada) following manufacturers’ protocols. In cultured cells, LDH release in the preservation solution was presented as a percentage of positive control (cells incubating with 2% Triton X-100). In sera or isolated hearts, LDH was expressed as the absorbance at 490 nm (OD_490_). Serum CK level was measured as a unit per liter (U/L) using Enzychrom Creatine Kinase Assay kit (BioAssay System, Hayward, CA, USA).

### Ethidium Bromide Staining

At the end of ex vivo preservation, heart organs were stained with ice-cold phosphate-buffered saline (PBS) containing 0.5 μg/mL of ethidium bromide (EB) for 20 min. After unbound EB was removed by extensive washing with ice-cold PBS, each organ was axially sectioned from the bottom to the top into four slices. The intensity of EB staining in the slices was visualized and photographed under UV light.

### Histological Analysis

After PBS perfusion, tissue samples were removed at necropsy and fixed in 10% buffered formaldehyde. Specimens were then embedded in paraffin, and sectioned for hematoxylin and eosin (H&E) staining. Graft injury was determined in H&E-stained sections by histological analysis, and was pathologically scored in a blinded fashion based on the severity of cardiac tissue damage under the microscopic view as: 0: normal cardiac tissue; 1: mild damage, indicated by perivascular injury; 2: severe damage, indicated by the presence of both perivascular injury and mild cardiac hemorrhaging; or 3: severe hemorrhaging and cardiac dilation.

### Trypan Blue Exclusion Assay

Cell viability was assessed by negatively staining with trypan blue, a cell membrane impermeable dye. In brief, a confluent monolayer of HUVECs or H9c2 cells (0.2 × 10^6^ cells/well) in 24-well plates was grown overnight, followed by incubation with 0.5 mL of HPG solution or UW solution at 4°C for 24 h. Cells were detached with trypsin-EDTA solution (Sigma-Aldrich Canada), and the viable/surviving cells (trypan blue negative) were counted using a TC10 automated cell counter (Bio-Rad Laboratories Canada, Mississauga, ON, Canada). The percentage of surviving cells was calculated as follows: % = (T_x_/T_0_) × 100, where T_x_ represented the total number of viable cells at an indicated time point, and T_0_ is the total number of viable cells in an untreated cell monolayer (0 h time point). The number of viable cells in each sample is presented as the average of at least three determinants.

### Evaluation of Cell Membrane Fluidity

The cell membrane fluidity of cultured HUVECs (1×10^6^ cells/mL) was estimated using a membrane fluidity kit following the manufacturer’s protocol (Marker Gene Technologies, Eugene, OR, USA) as described previously [51]. The ratio of eximer (E) at 480 nm to monomer (M) at 390 nm was calculated as an indicator of membrane fluidity, higher eximer levels being associated with more fluid membrane environments.

### Immunohistochemical Analysis

Myeloperoxidase (MPO), a biomarker of infiltrating neutrophils, in the sections of cardiac tissues was localized by a standard immunohistochemical method, and MPO^+^ infiltrates were semiquantitated as described previously in a blinded fashion [50, 51]; the number of MPO^+^ infiltrates per view for each graft was determined by averaging at least 20 nonoverlapping microscopic views in two serial sections.

### Measurement of Adenosine Triphosphate

The levels of adenosine triphosphate (ATP) in the solutions or cellular extracts were measured using an ATP determination kit following the manufacturer’s protocol (Invitrogen—Life Technologies Inc., Burlington, ON, Canada). In brief, HUVECs or H9c2 cells (1×10^6^ cells/well) were grown in culture medium in 6-well plates overnight, followed by exposure to HPG solution or UW solution (0.5 mL/well) at 4°C for 4 h. After collection of the solution/supernatant, the intracellular ATP was extracted by the incubation of the cell with 0.35 mL/well of Somatic Cell ATP Releasing Agent (Sigma-Aldrich Canada). Both extracellular ATP in the supernatant and intracellular ATP levels in each experiment were calculated based on the ATP standards determined in the same assay.

### Statistical Analysis

Data are presented as mean ± standard error of the mean (SEM). The statistical significance of the difference between two groups was determined by two-tailed *t*-test. One-way analysis of variance (ANOVA) or two-way with Tukey’s multiple comparison test was also used as appropriate for comparisons among multiple groups. Values of p ≤ 0.05 were considered statistically significant.

## Results

### Synthesis and Characteristics of HPGs

HPGs were synthesized by anionic ROMP [33]. The characteristics of the different HPGs used for this study was given in Table 1. The MW of HPG polymers ranged from 0.5 to 119 kDa with relatively low PDI (< 1.46), whereas HES in UW solution had Mn of 135.5 kDa and PDI of 1.86. The hydrodynamic radius (R_h_) of HPG polymers ranged from 0.66 nm to 5.05 nm, and 8.79 nm for HES. The degree of branching of the HPGs was in the range of 0.56 as determined by ^13^C NMR spectroscopy [35]. A comparison of chemical structure of HPG and HES was shown in Fig. 1.

**Table 1:** Molecular characteristics of HPGs used for the preparation of organ preservation solutions.

Polymer	M_n_ (g/mol)	PDI (M_w_/M_n_)	Hydrodynamic radius (R_h_, nm)	Osmolality (mOsm/kg)
HPG (0.5kDa)	500	1.27	0.66	369
HPG (1.0kDa)	998	1.41	1.04	340
HPG (3.5kDa)	3,300	1.21	1.23	297
HPG (8.7kDa)	8,700	1.46	2.30	296
HPG (25 kDa)	25,000	1.23	3.07	290
HPG (46kDa)	46,000	1.26	3.73	285
HPG (119kDa)	119,000	1.23	5.05	276
HES*	135,500	1.86	8.79	337

* HES was isolated from UW solution by dialysis with 3.5 k membrane.

The average MW (Mn), polydispersity index (PDI, Mw/Mn), hydrodynamic radius (R_h_) and solution osmolality were measured as described in the Materials and Methods section.

### Screening Different HPGs in Cold Preservation of Mouse Hearts in *Ex Vivo* and Optimization of Cold Preservation Solutions

Initial studies were designed to arrive at an optimal MW of HPG and its concentration for the cold preservation of organs. The protection of mouse hearts by HPG solution was compared to conventional UW solution *ex vivo*. For the initial screening, HPG (3% w/v) dissolved in UW solution devoid of HES and raffinose was used. Since the osmolality of aqueous HPG solutions varied widely with different MWs of HPG, especially for low MW HPGs [47], for this initial screening all the HPG solutions were prepared at a concentration of 3 wt%. The osmolality of the preservation solutions was listed in Table 1.

**Fig 2:**
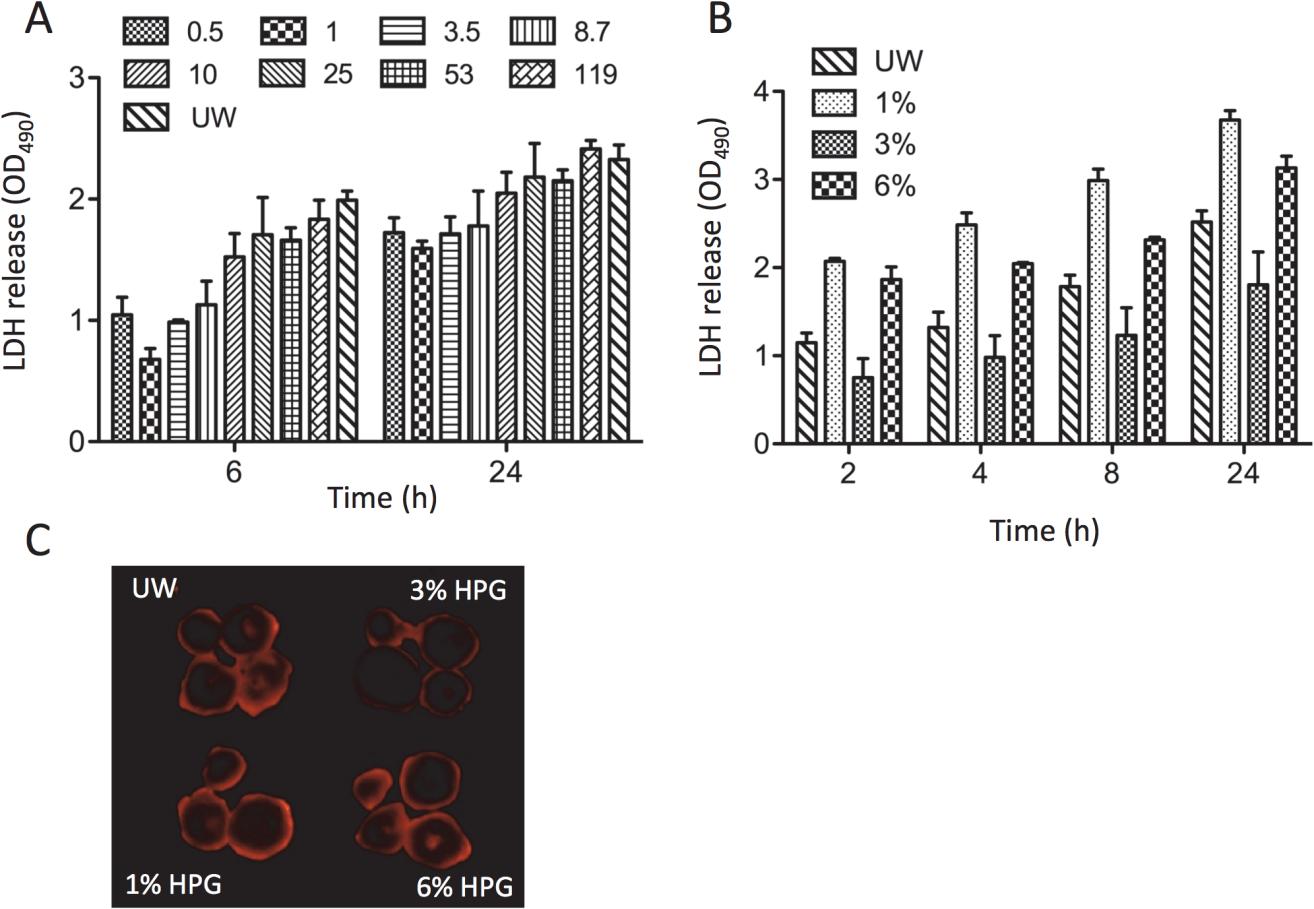
The size-dependent protection of mouse hearts from tissue damage at 4°C by HPGs. The hearts from naïve B6 mice were stored in HPG (3%, w/v) or UW solution (0.2 mL/organ) at 4°C for 24 h. (A) LDH release from mouse hearts during cold storage with different sizes of HPG (0.5–119 kDa). Data are presented as mean ± standard error of mean (SEM). Two-way ANOVA was used for statistical comparison between UW and HPG (vs. 0.5 kDa HPG, p = 0.008; vs. 1 kDa HPG, p < 0.0001; vs. 3.5 kDa HPG, p = 0.0001; vs. 8.7 kDa HPG, p = 0.0281; vs. 10 kDa HPG, p = 0.0976; vs. 25 kDa HPG, p = 0.5124; vs. 53 kDa, p = 0.0554; vs. 119 kDa, p = 0.8017. n = 4). (B) The tissue damage of the hearts during cold storage with different concentrations (1–6%) of 1 kDa HPG solution was determined by LDH release to the preservation solution. Data are presented as mean ± SEM. Two-way ANOVA was used for statistical analyses (p < 0.0001, HPG vs. UW, n = 4–7). (C) A representative image of EB-stained heart slices after 24-h cold preservation with 1 kDa HPG or UW solution. The fluorescence intensity in dead cells stained with EB was visualized with UV light.

The influence of MW of HPGs (0.5–119 kDa) on the protection of mouse hearts at 4°C in comparison to UW solution was given in Fig. 2A. The tissue damage of isolated mouse hearts preserved in HPG solution or HES-based UW solution (0.2 mL/organ at 4°C) was determined by LDH release from donor tissues at different time points. The lower the amount of LDH released, the better will be the organ protection. As shown in Fig. 2A, the hearts in 1 kDa HPG solution were mostly protected, indicated by the lowest levels of LDH release at both 6 h and 24 h time points. The levels of LDH release increased with increase in storage time. Statistical analyses (two-way ANOVA) suggest that there was no significant difference in LDH release between UW solution and any of 10–119 kDa HPG solutions (vs. 10 kDa, p = 0.0976; vs. 25 kDa, p = 0.5124; vs. 53 kDa, p = 0.0554; vs. 119 kDa, p = 0.8017. n = 4), while LDH release was significantly less in any of 0.5–8.7 kDa HPG solutions than in UW solution (vs. 0.5 kDa, p = 0.008; vs. 1 kDa, p < 0.0001; vs. 3.5 kDa, p = 0.0001; vs. 8.7 kDa, p = 0.0281. n = 4). Taken together, these data clearly indicated that only the smaller sizes (< 8.7 kDa) of HPGs were superior to HES as a colloid in cold preservation of mouse hearts, and there was a clear MW dependence.

Since, HPG 1 kDa solutions gave better protection than other molecular weights of HPGs, we next investigated the influence of concentration (1–6%) of HPG 1kDa in the protection of mouse hearts. As shown in Fig. 2B, the levels of LDH release were increased with increasing hypothermic storage time in all of groups; the amount of released LDH in either 1% or 6% of HPG solution was significantly higher than that in UW solution (p < 0.0001, two-way ANOVA, n = 3–4), but consistently, 3 wt% 1 kDa HPG based solution gave less LDH release from the mouse hearts compared to UW solution; the LDH level for HPG 1 kDa 3 wt% group was increased from 0.75 ± 0.21 at 2 h to 1.8 ± 0.38 at 24 h compared with 1.15 ± 0.11 at 2 h to 2.52 ± 0.12 at 24 h in the UW group (p < 0.0051, two-way ANOVA, n = 3–4). This observation was further confirmed by EB-staining of heart tissues. As shown in Fig. 2C, the intensity of EB staining of the hearts after 24 h incubation with the 1 kDa 3 wt% HPG solution was lighter than 1 or 6 wt% HPG solutions compared to UW solution. Based on these studies, it was confirmed that 1 kDa HPG at 3 wt% concentration (here after referred to as HPG solution) provided better protection than any other sized HPGs or UW solution for organs storage in hypothermia.

### Investigation of Functional Recovery and Tissue Damage of Cardiac Isografts

**Fig 3:**
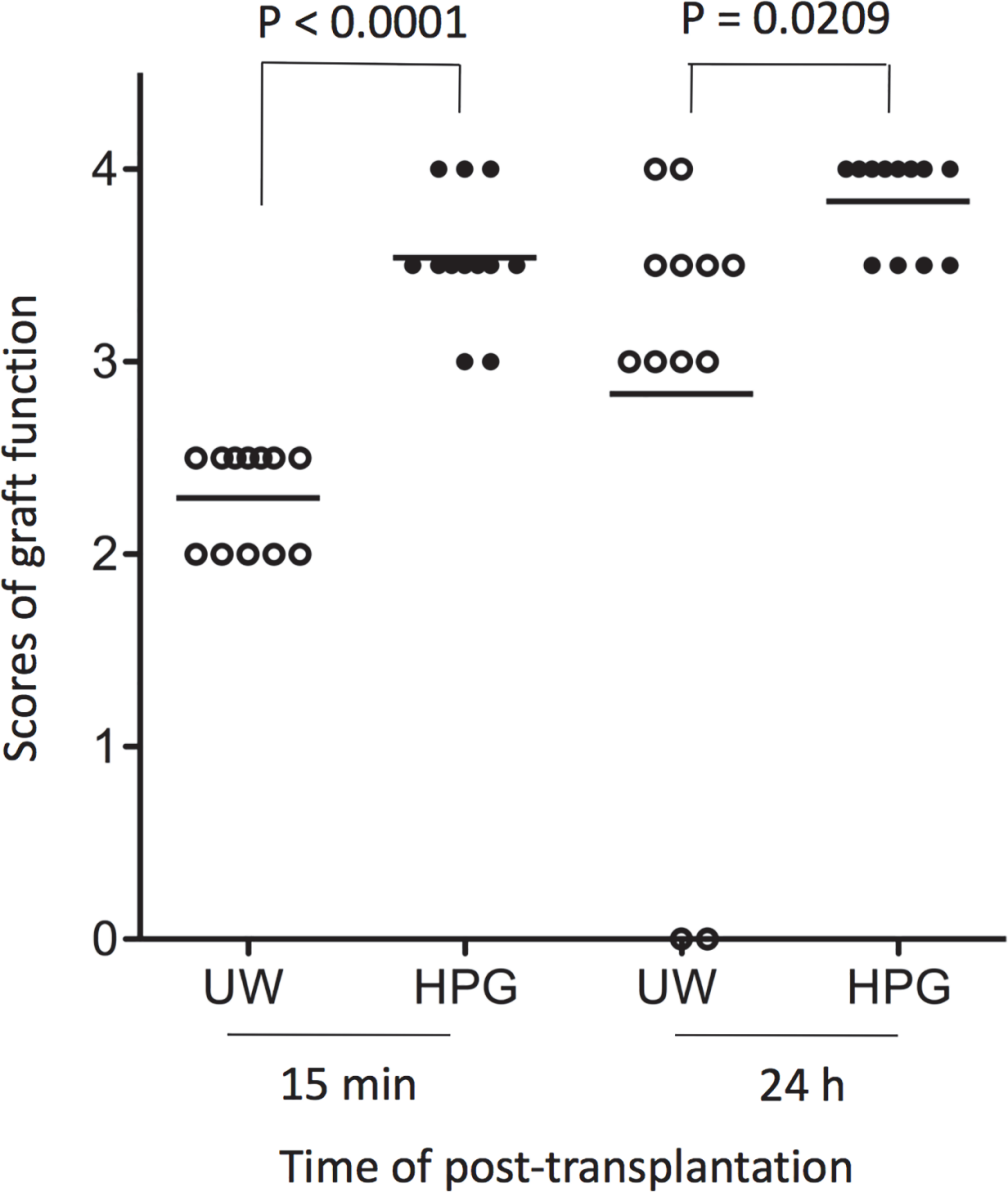
Cold preservation of donor hearts in HPG solution (1 kDa, 3%) enhances functional recovery after transplantation. Donor hearts were harvested from naïve B6 mice, and were stored in HPG or UW solution (0.5 mL/organ) at 4°C for 24 h. After transplantation to syngeneic B6 recipient mice, the graft function that was determined by the clinical score of graft contraction/beating was examined at both 15 min and 24 h post-transplantation. *Score* 4: normal contraction (equal to < 30 minutes of cold preservation in UW solution). At 15 min, p < 0.0001 (*t*-test, HPG vs. UW). At 24 h, p = 0.0209 (*t*-test, HPG vs. UW).

After confirming the enhanced protection of donor hearts by HPG solution over UW solution, we next investigated whether the protection can be translated to their functional recovery after transplantation. The relative viscosity and osmolality of HPG solution were measured as compared to UW solution, indicating that the viscosity of HPG solution was more than 2.5-fold lower than that of conventional UW solution (Table 2). To confirm if the superior prevention of heart tissue damage by HPG solution over UW solution could benefit to transplantation, the hearts from B6 mice after 24 h of cold storage at 4°C with HPG solution or UW solution (0.5 mL/organ) were heterotopically transplanted to syngeneic B6 mice. As shown in Fig. 3, the clinical score of graft function of heart grafts pretreated with the HPG solution was significantly higher than those stored in UW solution at both 15 min and 24 h after surgery, indicated by the mean score 3.542 in the HPG group compared to 2.292 in the UW group at 15 min (p < 0.0001), or 3.833 in HPG compared to 2.833 in UW (p = 0.0209) at 24 h. In the case of the UW solution group, 2 out of 12 grafts lost their function completely at the 24 h time point.

**Table 2:** Characteristics of the optimized HPG organ preservation solution in comparison to UW solution.

	HPG solution (3%, 1 kDa)	UW solution
Osmolality (mOsm/kg)	340	337
Relative viscosity at 4°C	1.375	3.514
Relative viscosity at 25°C	1.244	3.175

**Fig 4:**
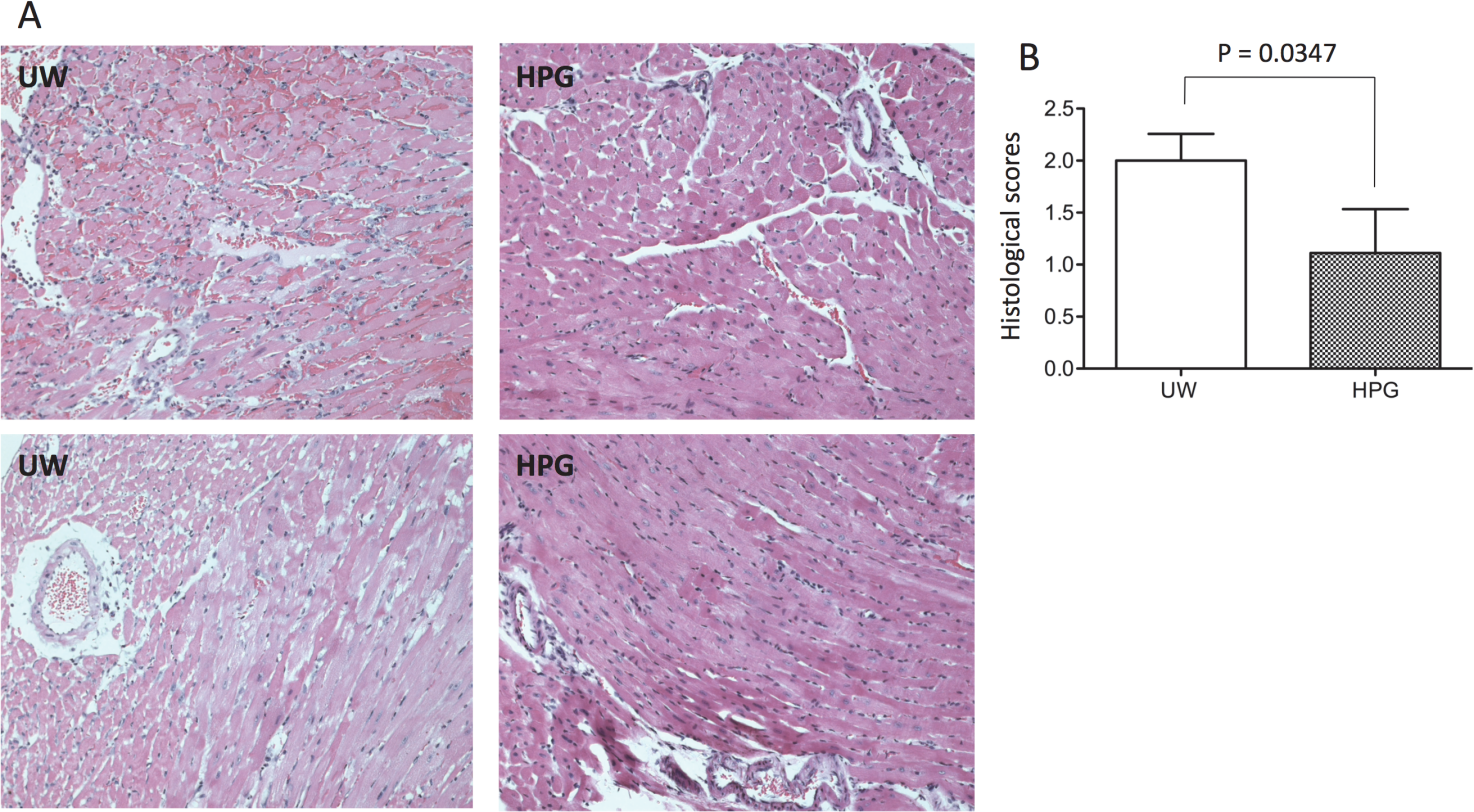
Cold preservation with the HPG solution reduces cardiac inflammation and cell death in heart transplants. Donor hearts were treated with prolonged cold preservation in HPG solution vs. UW solution and transplanted as described in Fig. 3. The grafts were harvested at 24 h after transplantation, and were formalin-fixed and paraffin-embedded. (A) The graft injury was examined in H&E stained sections. Data are presented as a typical image of light microscopy, showing perivascular inflammation and cardiaomyocyte necrosis. (B) Histological scores of the graft injury in HPG versus UW solution group. Data are presented as mean ± SEM in each group (p = 0.0347, *t*-test, HPG vs. UW, n = 9–10).

**Fig 5:**
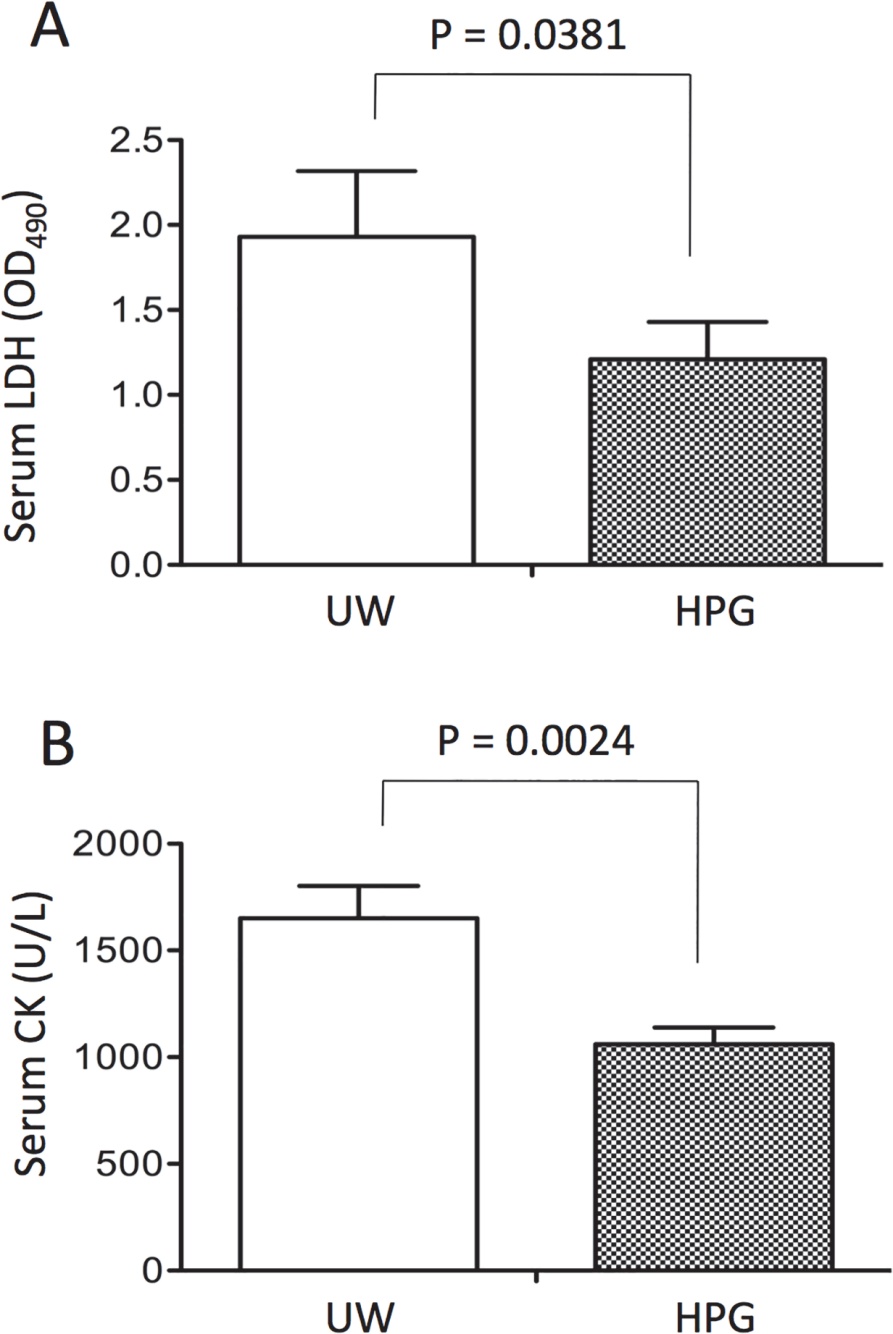
The severity of graft injury was determined by the release of LDH and CK from heart transplants to the serum. Sera were harvested from recipients at 24 h after transplantation, and serum levels of LDH (A) or CK (B) as a biochemical marker of cardiac graft injury were quantitatively measured using cytotoxicity detection kit. Data are presented as mean ± SEM of ten recipients in each group (LDH: p = 0.0381; CK: p = 0.0024. Two-tailed *t*-test, HPG vs. UW, n = 10).

To further confirm the better functional recovery of transplants in the HPG group, tissue injury and neutrophil infiltration in heart grafts were examined at 24 h after transplantation. Sections of heart grafts stained with H&E stain showed that hearts stored in HPG solution exhibited less perivascular inflammation and cardiomyocyte necrosis compared to the grafts stored in the UW solution (Fig. 4A). As shown by the semi-quantitative scoring of injury (Fig. 4B), the HPG group had a significantly lower score (1.111 ± 0.423, n = 10) compared to 2.0 ± 0.258 in the UW group (p = 0.0347, n = 9). These observations were further confirmed by the lower levels of both serum LDH and CK in HPG group, indicated by the fact that the absorbance values of serum LDL measurement in recipients in HPG group were 1.21 ± 0.22, significantly lower than 1.93 ± 0.39 in UW group (p = 0.0381, n = 12) (Fig. 5A), or 1060 ± 276 U/L of serum CK levels in HPG groups that were significantly lower than 1650 ± 530 U/L in UW group (p = 0.0024, n = 12)(Fig. 5B).

**Fig 6:**
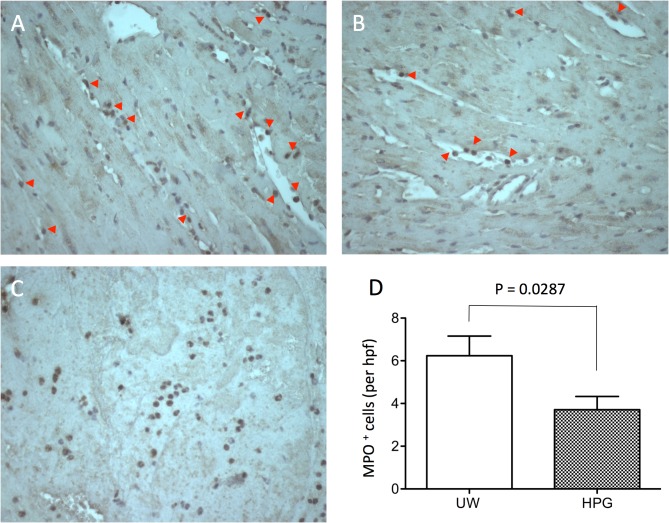
Cold preservation with the HPG solution reduces myeloperoxidase (MPO)-positive infiltration in heart transplants. MPO-positive cells in the sections of cardiac isografts were detected by immunohistochemical stain with anti-MPO antibody. The data are presented as a typical microscopic view in each group: (A) UW group; (B) HPG group; and (C) Positive control, blood clot. Red arrows point MPO-positive cells in the sections. (D) The number of MPO-positive infiltrates counted using a microscope under 400 × magnification (high-powered field, or hpf). The view was not overlapped and was randomly selected. At least 25 views from two separate sections were counted and averaged for each graft. Data are presented as mean ± SEM of six grafts in each group (p = 0.0287, two-tailed *t*-test, HPG vs. UW, n = 6).

Neutrophils are one of the first-responding inflammatory cells are the hallmark of acute muscle injury [53]. MPO-expressing infiltrates (activated neutrophils) in the cardiac sections were determined using immunohistochemical stain with anti-MPO antibody, and counted with a semiquantitative method. As shown in Fig. 6, the immunohistochemical stain of MPO^+^ infiltrates showed that graft sections of the HPG group had fewer infiltrating MPO^+^ cells (3.712 ± 0.615 cells/hpf, n = 5) than those of the UW group (6.237 ± 0.921 cells/hpf, n = 6) (p = 0.0287).

### Survival of Cardiac Allografts Preserved in HPG Solution

**Fig 7:**
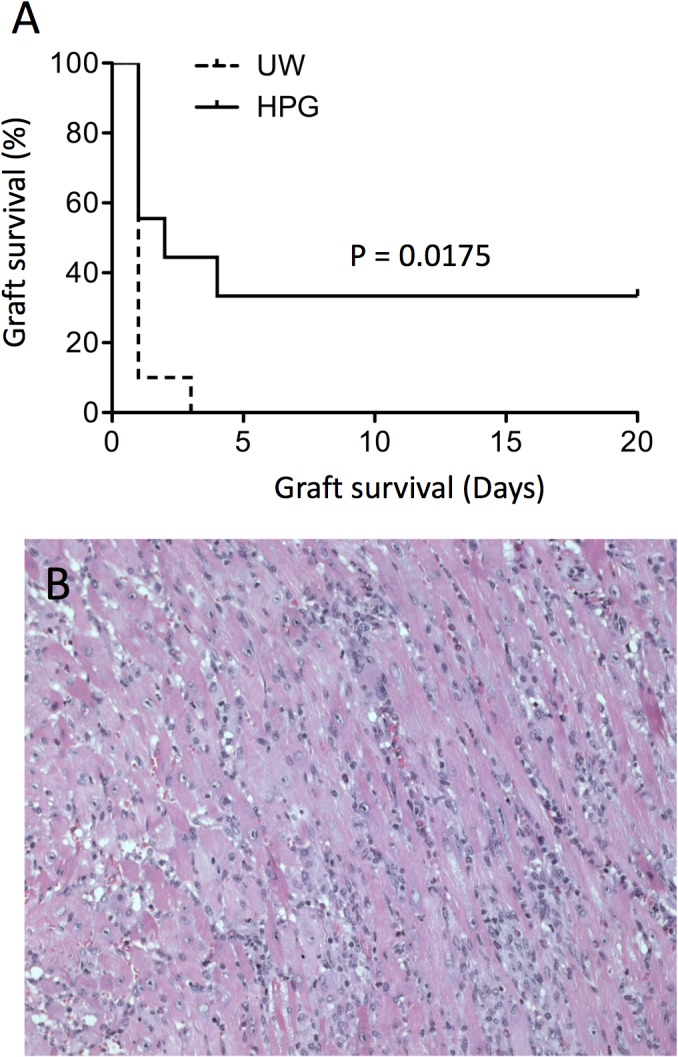
Cold preservation with the HPG solution prolongs survival of cardiac allografts. Donor hearts from naïve B6 mice were stored in HPG or UW solution (0.5 mL/organ) at 4°C for 24 h, and transplanted into allogeneic BALB/c mice that were treated with CsA daily. Graft survival was assessed by daily transabdominal palpation, and cessation of the graft beat was considered as graft failure. (A) Graft survival in HPG versus UW group (p = 0.0175, log-rank test, n = 9–10). (B) A typical microscopic view of H&E-stained sections of functioning grafts on day 20 post-transplantation.

In clinical transplantation, donor organs are mostly transplanted into allogeneic recipients, and these allografts survive under immunosuppressive therapy. To test the performance of the HPG solution in comparison to the UW solution in this setting, donor hearts from B6 mice were preserved with either HPG or UW solutions (5 mL/organ) at 4°C for 24 h. Stored hearts were heterotopically transplanted to allogeneic BALB/c mice that were receiving daily CsA treatment immediately after surgery. As shown in Fig. 7A, the population of allografts preserved in the HPG solution survived longer than those stored in UW. Only one transplant in the UW group survived 3 days, the rest of them failed within 24 h. In comparison, within the HPG group three of the grafts survived with function until the end of the experiment—for 20 days (p = 0.0175, Log-rank test), and only four out of nine transplants were rejected within 24 h. The functioning grafts on day 20 in the HPG group exhibited intact cardiac muscles but with severe cellular infiltration (Fig. 7B). These results suggest that ongoing optimization of the preservation solution may further improve the graft survival.

### Cold Preservation with HPG Solution Benefits Cell Survival *In Vitro*

**Fig 8:**
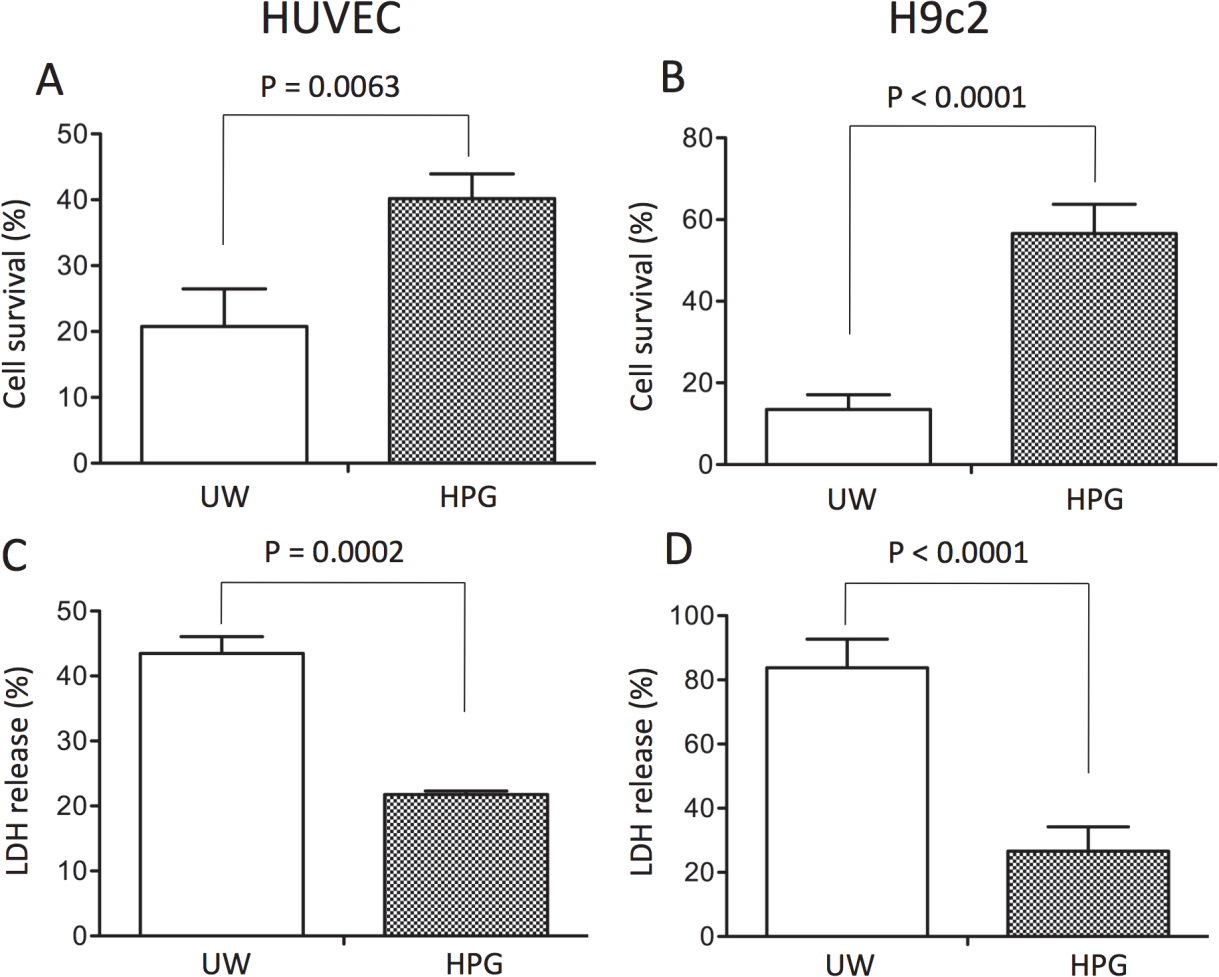
Cold preservation with HPG solution protects HUVECs or H9c2 cells from cell lysis at low temperature. Monolayers of HUVECs or H9c2 cells in 24-well plates were incubated with HPG versus UW solution at 4°C for 24 h. Cell survival was determined by a trypan blue exclusion assay. (A) HUVECs, p = 0.0063 (Two-tailed *t*-test, HPG vs. UW, n = 4). (B) H9c2 cells, p < 0.0001 (Two-tailed *t*-test, HPG vs. UW, n = 7). Cell death in the same cultures was confirmed by the measurement of LDH release. LDH in the preservation solution as a marker of cell lysis was measured and was calculated as a percentage of total LDH in a corresponding positive control (UW solution containing 2% Triton-100). (C) HUVECs, p = 0.0002 (Two-tailed *t*-test, HPG vs. UW, n = 4). (D) H9c2 cells, p < 0.0001 (Two-tailed *t*-test, HPG vs. UW, n = 12). Data are presented as mean ± SEM of four separate experiments in each group.

To compare the effects of the HPG solution with UW solution on hypothermic preservation at the cellular level, the impact of these solutions on survival of both cultured HUVECs and H9c2 cells at 4°C was determined. As shown in Fig. 8A, there were more surviving HUVECs exposed to the HPG solution (40.19 ± 3.77%) compared to the UW solution (20.75 ± 2.87%) (p = 0.0063, n = 4). The beneficial effect was further confirmed by the lower levels of LDH release in the HPG solution (21.76 ± 0.29%) compared to 43.46 ± 2.6% in UW solution (p = 0.0002, n = 4) (Fig. 8C). The LDH release from HUVEC cultures under normal 24 h culture conditions was approximately 21%, suggesting that the HPG solution might completely protect cultured human endothelial cells from cell lysis at 4°C over this period. In cultured H9c2 cells, similar protection of cells from death was seen after exposure to cold HPG solution as compared to UW solution (Fig. 8B and D).

### Cold Preservation with HPG Solution Maintains Intracellular ATP *In Vitro*

The loss of ATP supply in cells exposing to anoxia or hypothermia is a key initiator for the cascade of events, such as net Na^+^ influx and K^+^ efflux, and membrane damage and leakage, leading to necrotic cell death [54]. To investigate if the beneficial effect of HPG solution on cell survival at cold temperature was associated with an increase in intracellular ATP, both extracellular and intracellular ATP levels in the cells (both HUVECs and H9c2 cells) were measured after 4 h of hypothermic preservation with UW solution versus HPG solution. As listed in Table 3, the extracellular ATP, released from HUVECs after exposure to cold UW or HPG solutions, was not significantly different (137.14 ± 5.81 pmol vs. 130.04 ± 5.25 pmol, p = 0.5740), while the intracellular ATP (50.67 ± 1.17 pmol) of HUVECs in the HPG solution was significantly higher than that (43.0 ± 1.27 pmol) of those preserved with the UW solution (p = 0.0208); the ratio of extracellular to intracellular ATP in the HPG group was also significantly lower than that in the UW group (2.55 ± 0.05 vs. 3.18 ± 0.09, p = 0.0039). The beneficial effect of HPG solution on the maintenance of intracellular ATP was even more significant in H9c2 cells (Table 4); no leaking extracellular ATP was detected in these cells after 4 h of cold storage with HPG solution, whereas 403.11 ± 31.67 pmol were found in those with UW solution (p = 0.0011), and the intracellular ATP (798.97 ± 55.09 pmol) in HPG group was more than 2 times higher than that (307.73 ± 19.98 pmol) in UW group (p = 0.0346).

**Table 3:** Cold preservation with HPG solution maintains intracellular ATP pool in cultured HUVECs at 4°C.

	HPG solution	UW solution	P value (HPG vs. UW, n = 3)
Extracellular ATP (pmol)	130.04 ± 18.19	137.14 ± 20.11	0.5740
Intracellular ATP (pmol)	50.67 ± 4.03	43.0 ± 4.4	0.0208
Ratio	2.55 ± 0.17	3.18 ± 0.31	0.0039

**Table 4:** Cold preservation with HPG solution maintains intracellular ATP pool in cultured H9c2 cells at 4°C.

	HPG solution	UW solution	P value (HPG vs. UW, n = 6)
Extracellular ATP (pmol)	6.66 ± 13.73	403.11 ± 31.67	0.0011
Intracellular ATP (pmol)	798.97 ± 55.09	307.73 ± 19.98	0.0346
Ratio	0.05 ± 0.02	1.30 ± 0.06	0.0002

### HPG Solution Increases Cell Membrane Fluidity at Cold Temperature

**Fig 9:**
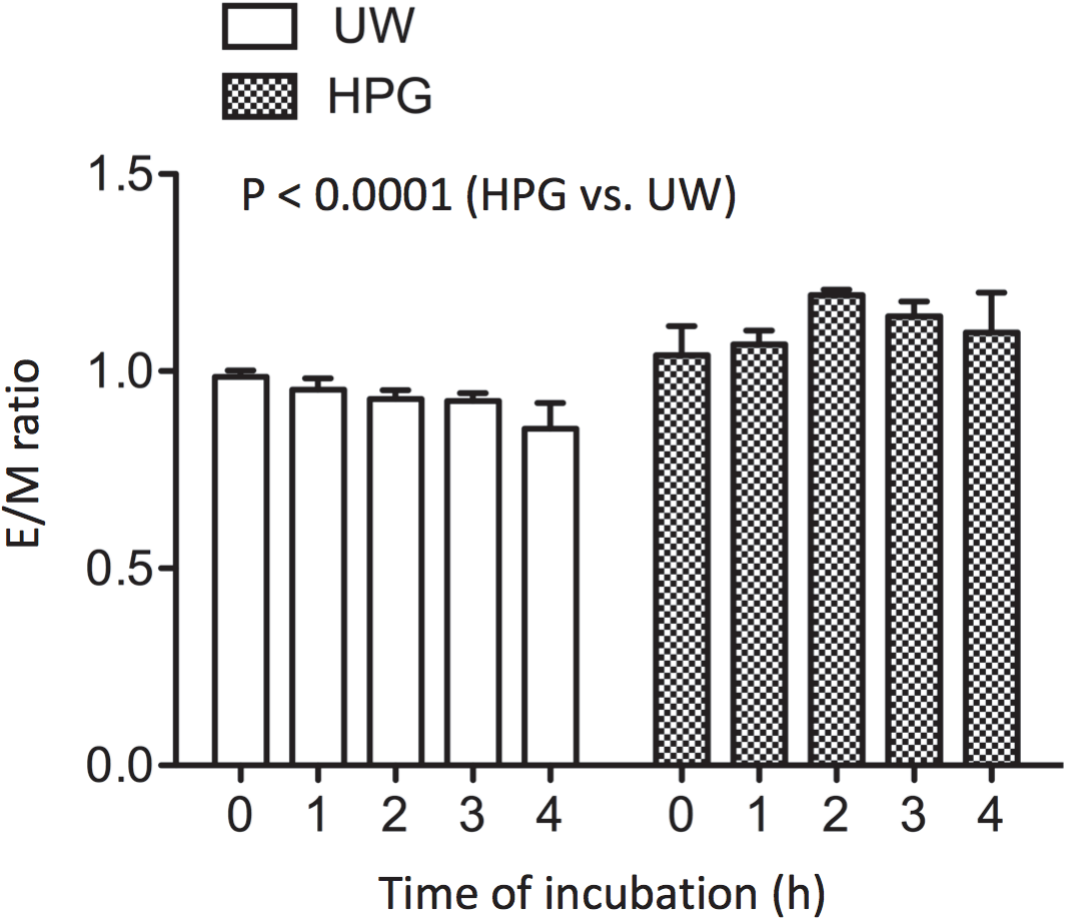
Cold preservation with HPG solution enhances cell membrane fluidity of HUVECs at low temperature. The cell membrane fluidity of HUVECs in HPG versus UW solution at 4°C was monitored by pyrene eximer formation for a period of 4 h. The ratio of eximer-to-monomer (E/M) was calculated as an indictor for the membrane fluidity at the various time points. Data are presented as mean ± SEM of five separate experiments (p < 0.0001, two-way ANOVA, HPG vs. UW, n = 5).

To investigate the possible mechanisms behind the better performance of HPG over UW solutions in the hypothermic protection from cell death and the maintenance of intracellular ATP in cultured cells, the influences of these solutions on membrane fluidity in HUVECs during 4 h of cold storage were compared. As shown in Fig. 9, there was a decreasing but not statistically significant trend in E/M ratio from 0.99 ± 0.02 at 0 h to 0.85 ± 0.07 at 4 h (p = 0.1887, one-way ANOVA, n = 3) in HUVECs after cold exposure in UW solution. However, the E/M ratio in these cells in the HPG solution remained unchanged in the period of study (4 h), indicated by 1.04 ± 0.07 at 0 h to 1.10 ± 0.1 at 4 h (p = 0.4647, one-way ANOVA, n = 3). Statistical comparison of the E/M ratio between these groups suggested that the E/M ratio was significantly higher in HUVECs in the HPG solution than those in the UW solution (p < 0.0001, two-way ANOVA).

## Discussion

HES-based UW solution is commonly used for both aortic *in situ* flush for heart transplants and *ex vivo* cold storage of kidney, liver and pancreas. Recently, in comparison with HTK solution in preservation of deceased-donor organs, UW solution was demonstrated to reduce graft loss in kidney transplants even beyond 12 months [55–57], and prolong graft survival in both liver and pancreas transplants [58, 59]. However, there are a number of donor organs that do not function well upon transplantation after the preservation with the UW solution [10], suggesting that an improved method or a new solution for maximally limiting this injury is needed, and perhaps by which increasing the donor pool by using expanded criteria or marginal donors could be possible [60]. The present study demonstrates, for the first time, that the replacement of HES and raffinose with HPG, a compact biocompatible polymer, in the UW solution enhances the protection of donor hearts from cold ischemic injury and cultured cells (HUVECs and H9c2 cells) from cold-induced cell death. Importantly, the cytoprotection of low MW HPGs was found to be superior to that of the higher MW HPGs.

When the blood in donor organ is replaced with a preservation solution for the cold preservation prior to transplantation, the vascular endothelium is first structure to get exposed to artificial environment. Indeed, loss of endothelial integrity represents a primary event in cold preservation-related graft injury in various organ transplants [61–64]. It has been proposed that cold injury impairs the barrier function of the endothelium, thus leading to parenchymal edema and hemorrhage following reperfusion, and early graft dysfunction [65, 66]. HES is included in UW solution as a key component for the prevention of both interstitial edema and endothelium swelling [9, 10]. To our knowledge, this is the first study to show the superiority of HPG (0.5–8 kDa) over HES (∼250 kDa) in the reduction of tissue damage during the static cold storage of mouse hearts (Fig. 2A), and 1 kDa HPG (3%)-based solution is superior to UW solution with similar osmolality (Fig. 2B, Table 2). The beneficial effects of replacing linear HES with small, compact 1 kDa HPG polymer on the prevention of organ injury during cold preservation are also evidenced by better functional recovery, less perivascular inflammation and cellular infiltration, graft tissue damage and prolonged graft survival after transplantation. However, the mechanisms for the superior protection of HPG over HES in UW solution are not investigated as of yet, and one has to acknowledge the limitations of this study. First, it is not clarified yet whether or not the size of HPG or the solution osmolality or even both are critical for the cold protective activity of HPG solutions. A lower MW HPG is more practical than large MW HPGs for new organ preservation development, and also compared to glucose, 0.5 kDa or 3 kDa HPG, 1 kDa HPG exhibits the best biocomptability profile under similar solution osmolality in a rat model of peritoneal dialysis [47, 48]. Second, the present study does not provide direct evidence supporting the beneficial effect of HPG solutions protecting vascular endothelium from tissue damage during cold storage of donor hearts and after transplantation, however, our *in vitro* data show that the HPG solution provides better protection of both cultured endothelial cells and cardiomyocytes from necrosis as compared to UW solution (Fig. 8).

HPG (0.5–8 kDa) and HES in UW solution are totally different polymers; HPG is a compact and small polyglycerol, whereas HES is a linear and large polyglucose, indicating that the different physical and chemical properties may exert different interactions of these two polymers with cell surface. Probably large and linear HES has high affinity binding to the cell as compared to small compact HPG. Cell membranes are suggested as a primary site of cold-induced injury [67]; following exposure to a cold temperature, cell membranes are destabilized by the change of membrane structure, such as modifications of specific lipid-protein interaction, phospholipid asymmetry and lipid composition, as well as potentially a local membrane transition from the liquid-crystalline phase to the lethal gel phase [68–70]. These changes eventually lead to decrease in membrane fluidity that has been demonstrated as a key factor for the susceptibility to cold-induced injury in many types of cells [71, 72]. This is further supported by our previous observations [50, 51], showing that clusterin, a chaperone protein, prevents the reduction of membrane fluidity at hypothermia, and reduces cold ischemic injury. The membrane fluidity in human endothelial cells following cold preservation with HES-based UW solution for 4 h remains significantly lower than equivalently treated cells in the HPG solution in which the membrane fluidity remains unchanged (Fig. 9), suggesting that HPG but not HES may have an uncharacterized ability to stabilize the cell membrane in hypothermia. Other differences in the way HES and HPG interact with cell membranes have been reported, for instance HES induces RBC aggregation [19], whereas HPG does not [41], implying that these two polymers interact with the components of the cell membrane differently. It has been shown that the amount of HPG adsorbed to cell surface is much lower than linear polymers [49]. This suggests that cell-surface adsorption of HPG may not be reason for its better performance. Further investigations are warranted to understand the different interactions of HPG versus HES with the cell membrane components (proteins and lipid) and resultant changes in the conformation/organization of the cell membrane in hypothermia.

In cases of cold organ preservation of organs or cells—severe O_2_ limitation and hypothermia, imbalance of ATP supply to its demand and destabilization of cell membranes immediately occur [54, 73], resulting in loss of ion-transporting system activity, such as Na^+^ influx and K^+^ efflex, catastrophic membrane failure and cell death with membrane rupture [54]. There are two mechanisms by which the intracellular ATP pool can be depleted during cold storage of an organ: ATP leaking due to the membrane damage, and low rates of ATP production because O_2_ and substrates are restricted, and the activity of enzymes (i.e. ATPase) decreases at cold temperature. As demonstrated, HPG was able to maintain the membrane fluidity at 4°C, by which the ATP leaking could be prevented and O_2_ is still efficiently transported across the plasma membrane to enable ATP synthesis. Both of these two factors may help to prevent cell damage. Although the myocardiac ATP levels of donor hearts preserved with HPG solution in comparison to UW solution have not been examined for ethical reasons—limiting animal experimentation if possible—the intracellular ATP in both HUVECs and H9c2 cells after hypothermic preservation showed increased levels compared to UW solution. There was also no ATP leaking from H9c2 cells in HPG solution compared to those in UW solution (Table 4). These data imply that HPG is superior to HES as a colloid for cell membrane protection at cold temperature for mouse hearts. Although the molecular mechanisms for the superiority of small HPGs over HES in a cold preservation solution against cold ischemia injury in both mouse hearts and cultured cells were not investigated in this study, based on the evidence in literature and some preliminary data presented here, it is hypothesized that because of the low affinity binding to the cell surface, small compact HPG polymer is a more compatible colloid for stabilizing cell membrane at cold temperature, which leads to an increase in intracellular ATP and benefits cell survival. In addition to that, the omission of raffinose in the preservation solution may have beneficial effect as raffinose-free UW solution has been shown to increase the function of canine hearts after extended preservation [74], and raffinose at as low as 10 mM suppresses endothelial cell growth [75]. Together with our data, these studies suggest that raffinose-free HPG solution may have additional beneficial effect on endothelium homeostasis.

Reducing cold ischemic injury to donor organs by optimizing cold preservation is a precondition for the success of organ transplantation and could benefit the increased use of marginal donors and non-heart beating donors [60]. Data from this study clearly show that the HPG solution in comparison to the standard UW solution is more effective in reducing cold ischemic injury in donor hearts. However, one has to acknowledge that the present study was performed using a mouse model and cultured human cells so results may not be the same as for transplants in patients. Further investigations are needed to test the utility of HPG solution in other cell/organ types, and using larger animals are warranted before making safe conclusions.

In addition, HES is a highly viscous agent; the UW solution causes aggregation of RBCs [19], and inhibits the collagenase digestion of human pancreas for islet isolation [76]. Since HPG produces a lower viscosity solution (Table 2) owing to its lower intrinsic viscosity, it is expected to help reduce organ perfusion-related injury. The intrinsic viscosity of the HPG is similar to proteins [38] and does not expected increase the viscosity tremendously in solution in a concentration-dependent manner. The highly reduced viscosity of HPG solution is anticipated to decrease the resistance and increase the flow compared to UW solution. We have previously shown that unlike linear polymers, such as HES and PEG, HPGs did not increase the cell aggregation, which would be beneficial in terms of their use as a flush solution [41]. Hence, the HPG solution may be a promising alternative to the UW solution for cold preservation of donor organs, tissues or cells in transplantation.

## Conclusions

In the present study we investigated the potential of HPG as a colloidal substitute in an organ preservation solution. Using our initial screening studies, we determined that HPG 1 kDa at 3 wt% concentration is superior in preventing in organ injury compared to other molecular sizes and concentrations of HPG. There was clear MW dependence. HPG solution showed superior performance compared to UW solution in terms of functional recovery, graft function in both isografts and in allografts in mouse models. HPG solution produced better survival in cultured cells in cold temperature. The maintenance of the intercellular ATP and preservation of the membrane fluidity of the cells may be responsible for the enhanced protection offered by HPG solution. Our studies demonstrate that HPG based solutions are superior to HES based UW solution in many aspects and has the potential to replace current cold preservation solutions in the protection of donor organs, tissues or cells for transplantation.
